# Taxonomy of the genus *Metolinus* Cameron (Coleoptera, Staphylinidae, Staphylininae, Xantholinini) from China with description of three new species

**DOI:** 10.3897/zookeys.112.1138

**Published:** 2011-06-24

**Authors:** Yu-Lingzi Zhou, Hong-Zhang Zhou

**Affiliations:** *Key Laboratory of Zoological Systematics and Evolution, Institute of Zoology, Chinese Academy of Sciences, 1 Beichen West Rd., Chaoyang District, Beijing, 100101, P. R. China*; *Graduate University of the Chinese Academy of Sciences, 19A Yuquan Rd., Shijingshan District, Beijing, 100049, P. R. China*

**Keywords:** Coleoptera, Staphylinidae, Xantholinini, *Metolinus*, China, new species, identification key

## Abstract

This paper studies the taxonomy of the genus *Metolinus* Cameron, 1920 (Coleoptera: Staphylinidae, Staphylininae, Xantholinini) from China and describes three new species: *Metolinus xizangensis* **sp. n.** from Xizang (Tibet), *Metolinus emarginatus* **sp. n.** fromSichuan, and *Metolinus binarius* **sp. n.** from Yunnan. The Chinese fauna of the genus is thus increased to 8 species in total. A key to eight Chinese species is provided. Female genital segments and other important morphological characters are illustrated in line drawings for the new species as well as *Metolinus shanicus* Bordoni, 2002 and *Metolinus gardneri* (Cameron, 1945). The text also provides color plates with habitus photographs and a map to show the species’ geographical distribution pattern. The type specimens of the new species are deposited in Institute of Zoology, the Chinese Academy of Sciences (IZ-CAS).

## Introduction

The tribe Xantholinini is a large rove beetle group of more than 75 genera and was considered currently by most taxonomists as one of the six tribe level taxa in the subfamily Staphylininae (Coleoptera: Staphylinidae) (Newton et al 2000, Assing 2000, Herman 2001, Zhou 2005). The genus *Metolinus* is one of those genus-level taxa belonging to the tribe Xantholinini. It was erected by Cameron (1920) and included originally two species: *Metolinus basalis* Cameron, 1920 and *Metoponcus leucocnemis* Kraatz, 1859. Subsequently, Blackwelder (1952) designated *Metoponcus leucocnemis* as the type species of the genus. According to Herman (2001), the genus included totally 14 species which were described by Kraatz (1859), Bernhauer (1915), (Cameron (1920, 1932, 1933, 1936, 1937, 1950), Scheerpeltz (1957), Coiffait (1977) and Last (1980). In the last decade, (Bordoni (2002, 2003b, 2003c, 2004, 2005a, 2005b, 2006, 2009b, 2009c, 2010a, 2010b) made enormous contributions to the knowledge of the genus and published 89 additional species so that the total number of species was increased to 116 for the world fauna.

**Figure 1.**
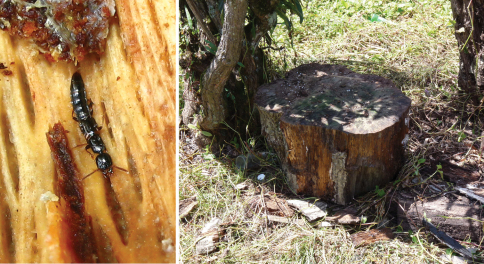
Habitat and feeding photo of *Metolinus shanicus*Bordoni, 2002 (photos by Xinlei Huang).

Individuals of the genus *Metolinus* occur mostly in rotting wood under bark and thus are assumed saproxylobionts (Fig. 1). Available label data, our own collecting experience, and the morphology of the genus (i. e. compressed body shape, expanded protarsi) support this observation and confirmed that most *Metolinus* species were saproxylic. These biological characteristics might explain their rarity in collections; obtaining specimens was difficult and mainly achieved by methods targeting microhabitats on or near the ground.

Before this study, five species ofthe genus *Metolinus* were recorded to occur in the territory of China: *Metolinus planulatus* (Sharp, 1889), *Metolinus gardneri* (Cameron, 1945), *Metolinus hayashii* Bordoni, 2002, *Metolinus shanicus* Bordoni, 2002, and *Metolinus yunnanus* Bordoni, 2002. These Chinese species were all detected by Bordoni (2002). He described three species from China and transferred the other two from *Leptacinus* Erichson, 1839 to *Metolinus* till then. Assing (2009) published one additional Chinese species, *Metolinus parvioculatus* Assing, 2009, which was synonymized by Bordoni (2009a) and is now a synonym of *Mahavana watanabei* Bordoni, 2009.

This paper studies the taxonomy of the genus *Metolinus* Cameron, 1920 from China and describes three new species: *Metolinus xizangensis* sp. n. from Xizang (Tibet), *Metolinus emarginatus* sp. n. fromSichuan, and *Metolinus binarius* sp. n. from Yunnan. The number of Chinese species is thus increased to eight species in total. A key to these Chinese species is provided. Female genital segments and other important morphological characters are illustrated in line drawing for the species including the new ones and *Metolinus shanicus* Bordoni, 2002 and *Metolinus gardneri* (Cameron 1945). The text provides also color plates to show beetle habitus and a map to show the geographical pattern of species distribution (Fig. 15).

## Materials and methods

Specimens were relaxed in warm water (60 ºC) for about 5−8 hours, then cleared in 10% KOH for 5 minutes, and transferred in 75% alcohol. Cleared specimens were dissected to observe morphological details of mouthparts, abdominal segments VIII and sexual genital segments and the aedeagus. After examination, the body parts were stored permanently in glycerin for future studies. Observations and drawings were done under a compound microscope (ZEISS Stemi 2000-C). The specimens used in this study, including the types of the new species, are deposited in the Institute of Zoology, the Chinese Academy of Sciences (IZ-CAS).

**Figure 2.**
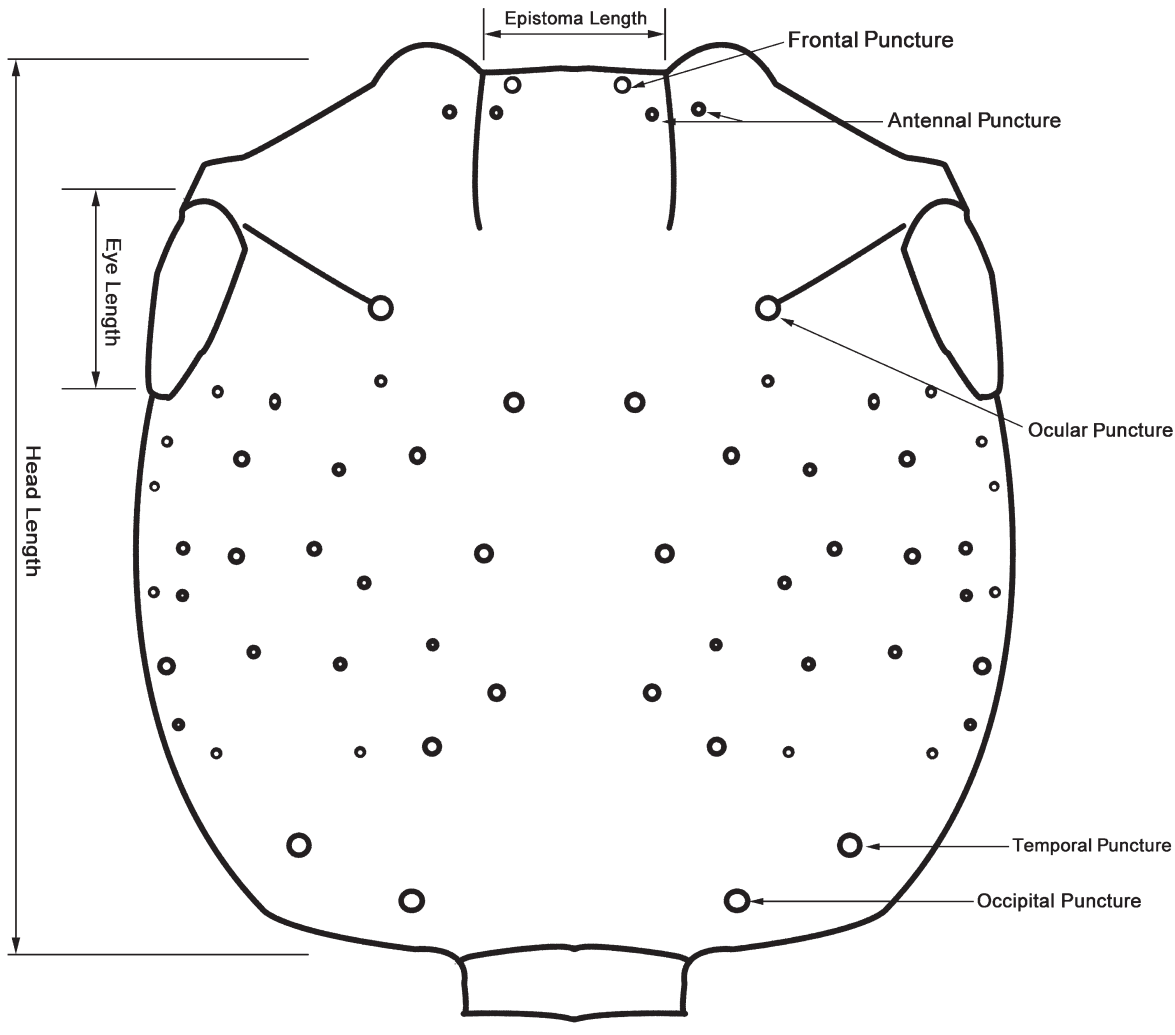
Chaetotaxy of head and measuring scheme of head (*Metolinus emarginatus* sp. n.) in dorsal view.

**Figure 3.**
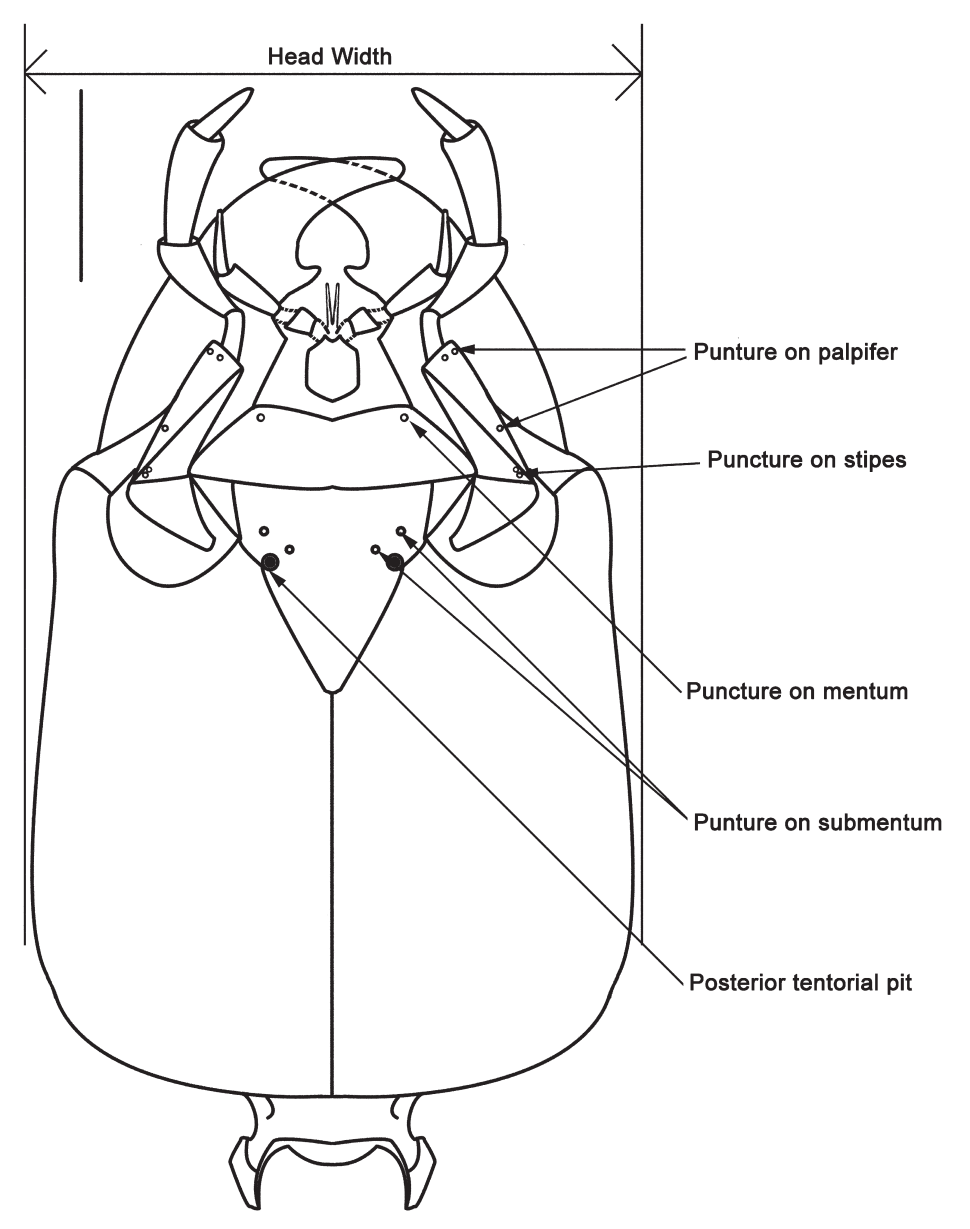
Characteristic punctures and measuring scheme of head (*Metolinus xizangensis* sp. n.) in ventral view.

**Figure 4.**
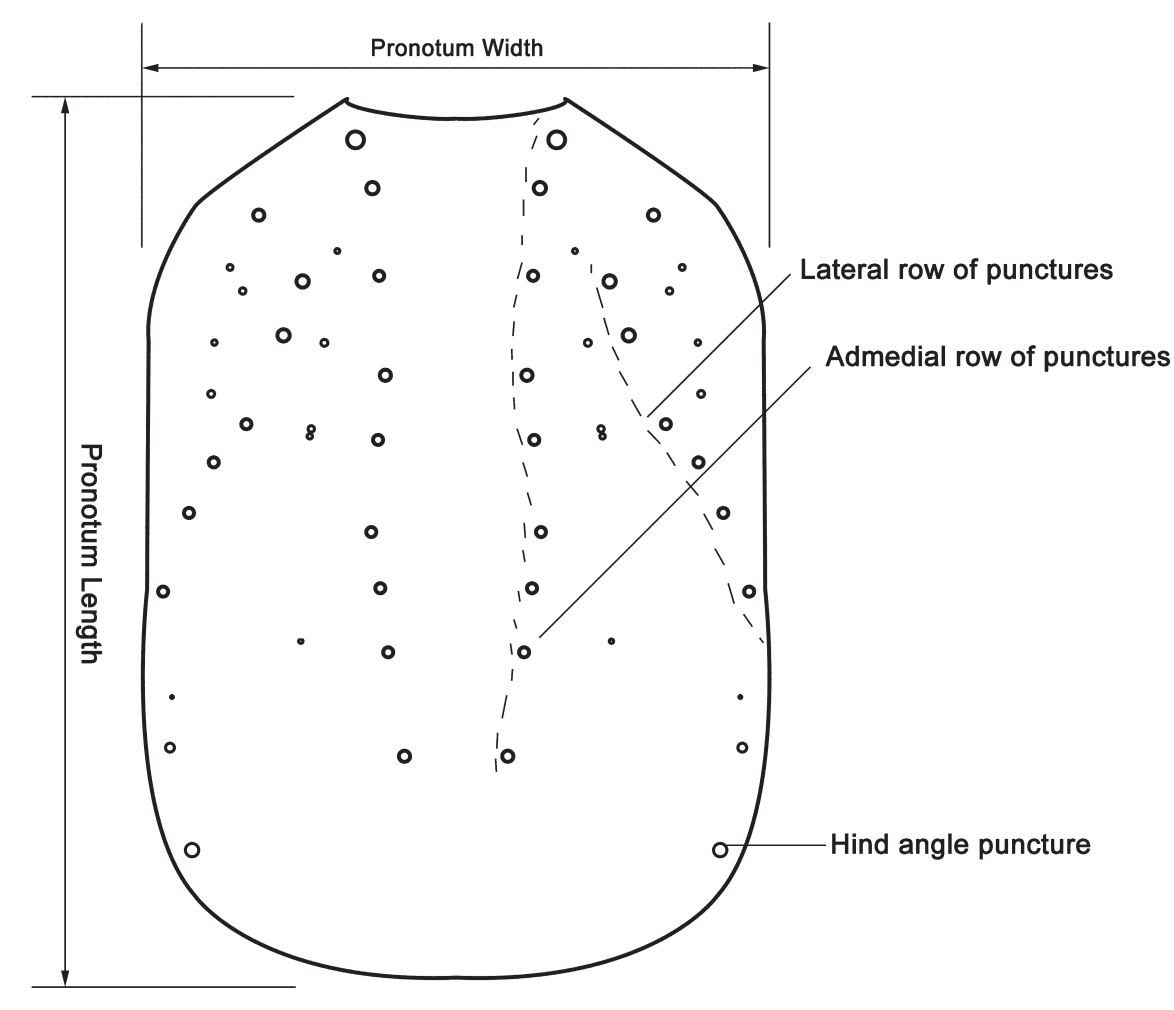
Puncture pattern and measuring scheme of pronotum (*Metolinus emarginatus* sp. n.) in dorsal view.

**Figure 5.**
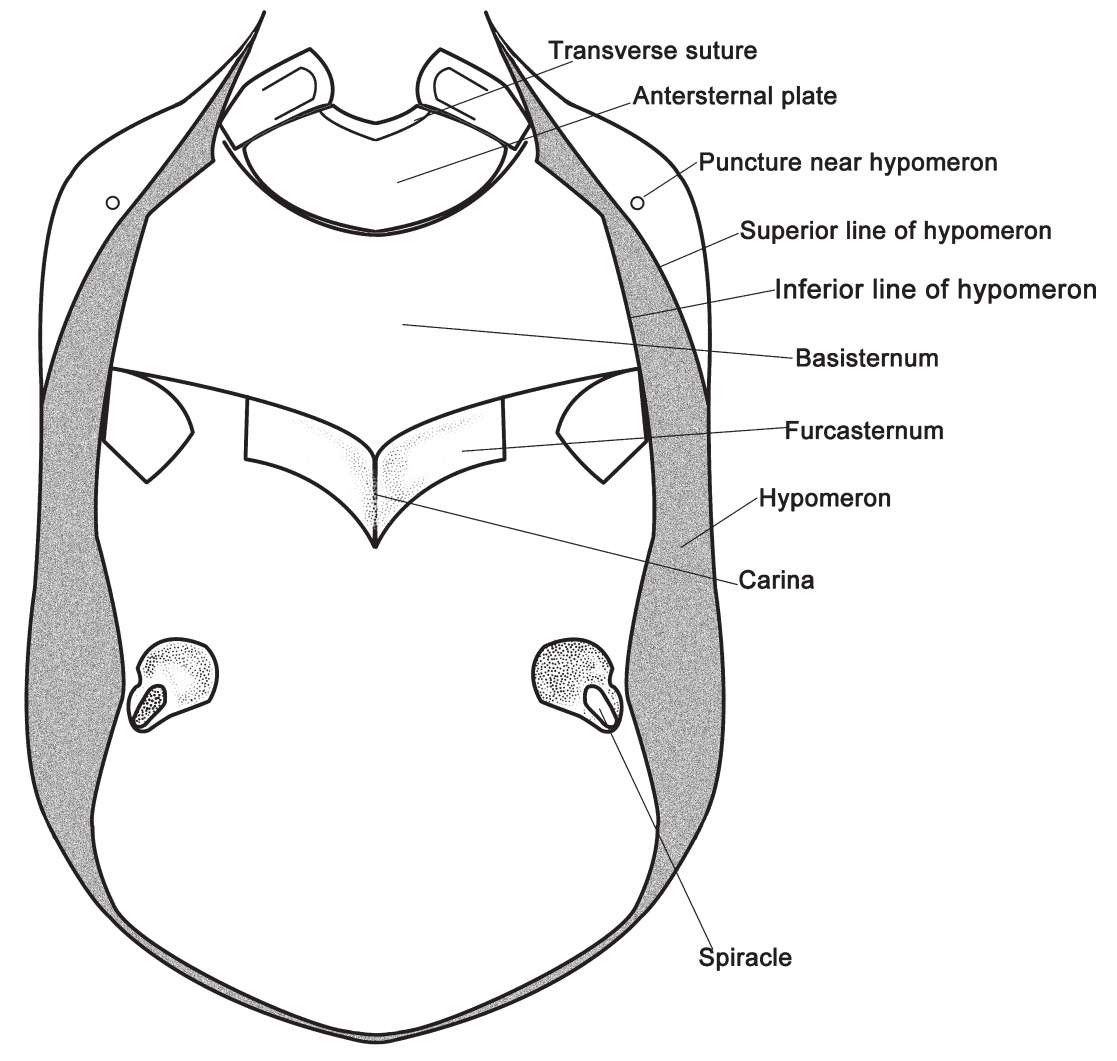
Schematic ventral view of prosternum (*Metolinus xizangensis*sp. n.).

**Figure 6.**
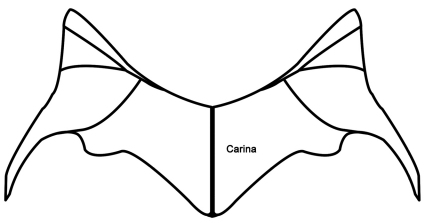
Schematic ventral view of mesoventrite (*Metolinus xizangensis*sp. n.).

**Figure 7.**
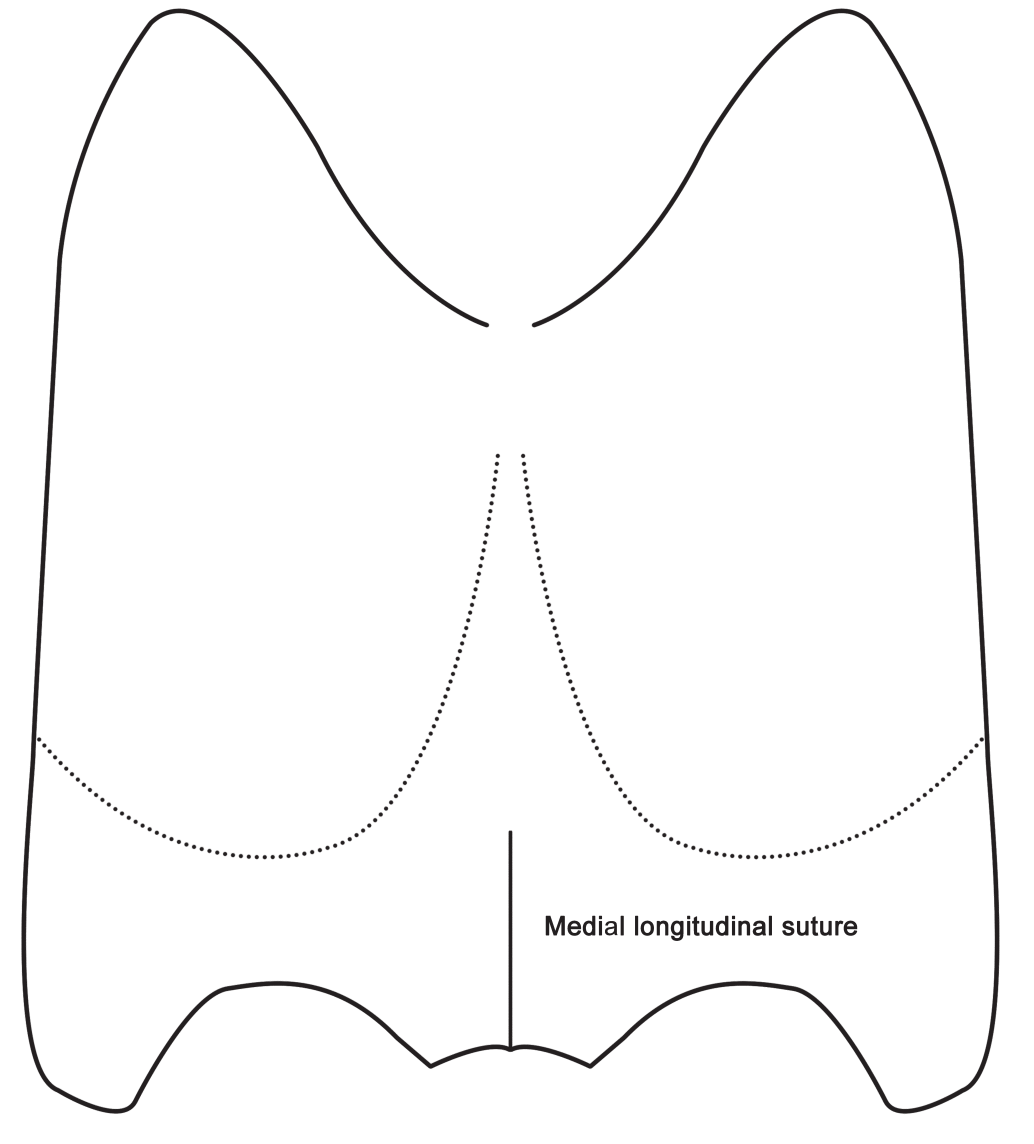
Schematic ventral view of metaventrite (*Metolinus xizangensis* sp. n.).

**Figure 8.**
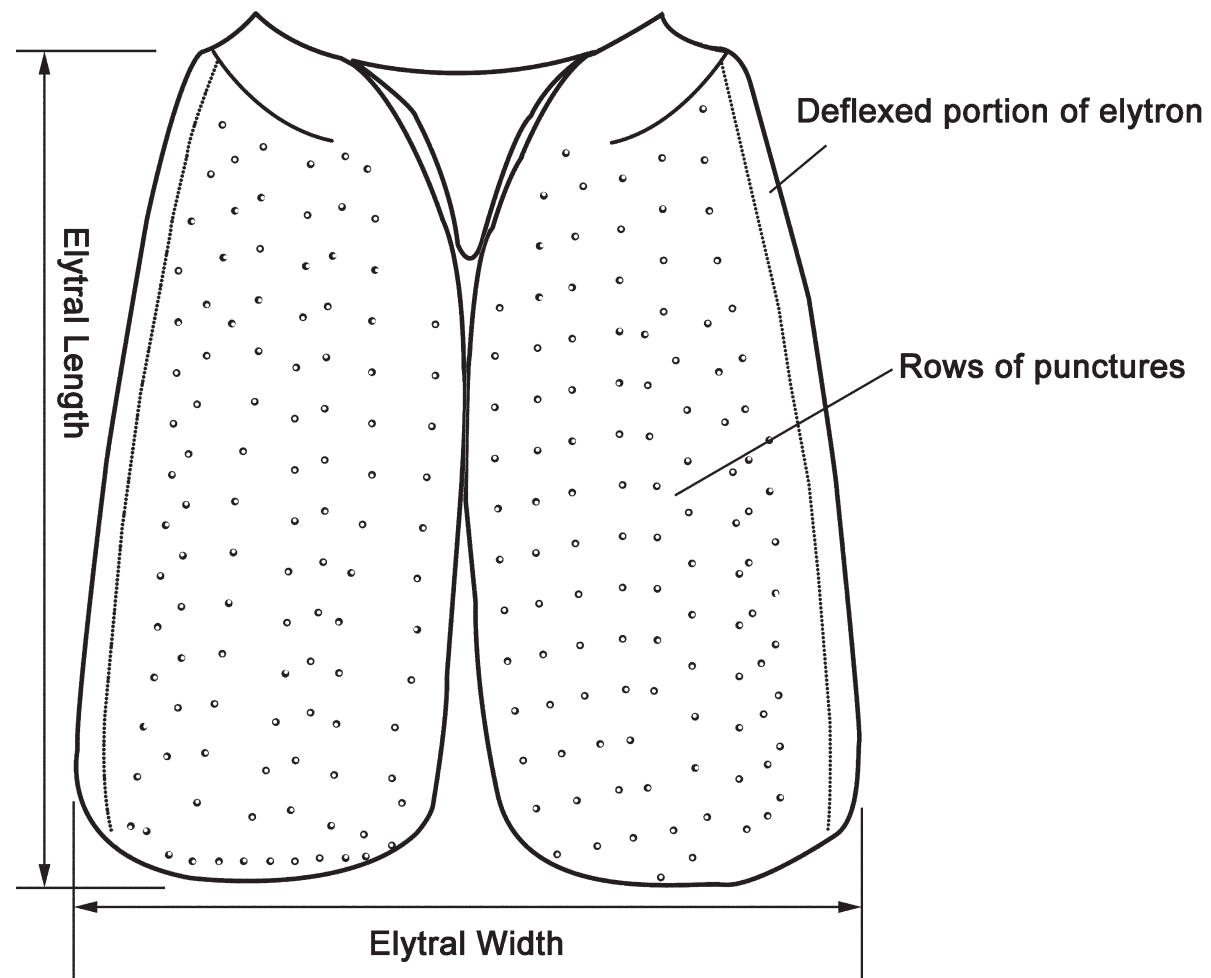
Puncture pattern and measuring scheme of elytra (*Metolinus emarginatus* sp. n.) in dorsal view.

**Figure 9.**
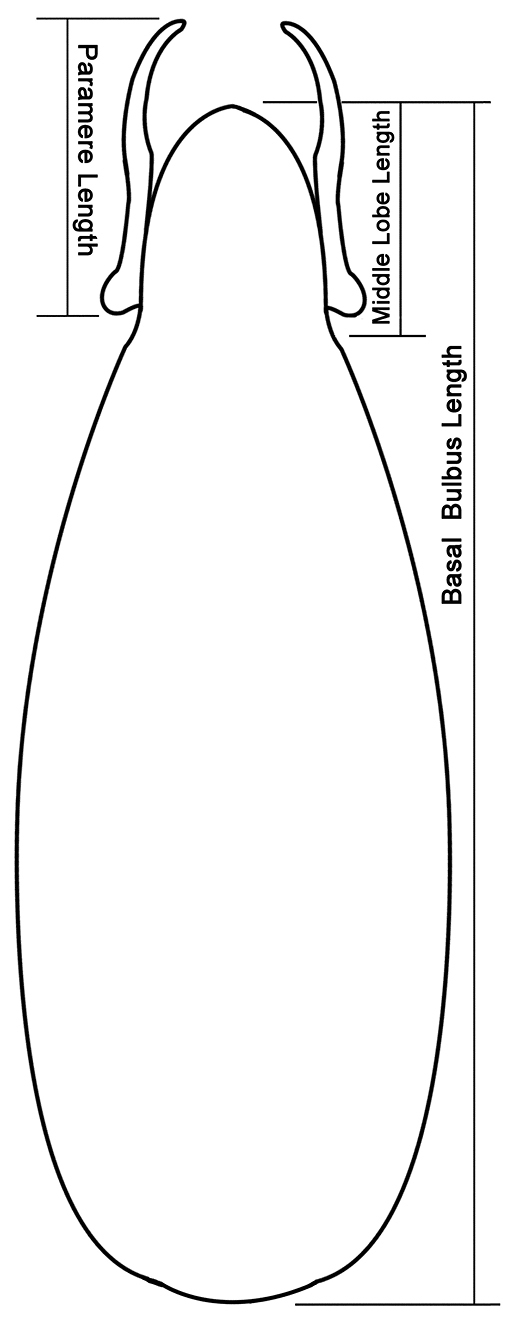
Measuring scheme of aedeagus (*Metolinus binarius*sp. n.) in dorsal view.

In describing morphological features, we followed Smetana (1982), Naomi (1990), Smetana and Davies (2000), Bordoni (2002), Gusarov (2002) and Solodovnikov and Newton (2005). In addition, schematic line drawings were employed to avoid confusions in describing and measuring of some important body parts and diagnosing important taxonomic characters (Figs 2–9). Measurements were done with the aid of an eyepiece micrometer under the compound microscope ZEISS Stemi 2000-C (Germany) and were given in millimeter (mm) in the text. The following abbreviations were used throughout the paper:

HLhead length (from apex of epistoma to neck constriction);
            

HWhead width (maximal, including eyes);
            

PLpronotum length (along mid line);
            

PWpronotum width (maximal);
            

ELelytral length (from acute humerus to most distal apical margin; best taken from lateral view of the elytron);
            

EWcombined width of both elytra (maximal, when elytra closed along suture).
            

Color photographs were captured with a Nikon D300 and the final deep focus images were created with the stacking software Helicon Focus 3.10. The species distribution database was compiled with Microsoft Excel and is based on the data of published records as well as label data of available specimens. The distribution map was produced with the aid of ArcGIS 8.3.

## Taxonomy

### Genus Metolinus

Cameron, 1920

http://species-id.net/wiki/Metolinus

Metolinus Cameron 1920: 147; Cameron 1932: 4 (characters; key to species of British India); Scheerpeltz 1933: 1298 (world catalog supplement); Shibata 1983: 68 (checklist of species of Japan); Herman 2001: 3703 (world catalog); Bordoni 2002: 337 (revision of oriental region; characters; key to species); Smetana 2004: 691 (Palearctic catalog); Bordoni 2005b: 539 (revision of Australia; characters; key to species); Bordoni 2007: 71 (catalog).

#### Type species.

*Metoponcus leucocnemis* Kraatz, fixed by subsequent designation by Blackwelder 1952: 241.

#### TDiagnosiss.

The genus *Metolinus* Cameron can be distinguished from all other genus level taxa within the tribe Xantholinini by the following characters: a) body nearly compressed, small to medium sized (3–8 mm), rarely larger (8–10 mm); b) head subquadrate or subrectangular (Fig. 2), often with microsculpture of microstriae (rarely of polygonal reticulum) and sparse medium punctures; c) frontal furrow often short or not obvious, ocular grooves distinct; d) penultimate segment of maxillary palpi and labial palpi distinctly longest, ultimate one slender and subaciculate (Fig. 3); e) pronotum with admedian and lateral row of punctures (Fig. 4); f) antesternal plate integrated (Fig. 5); g) superior line of hypomeron bending towards the prosternum before anterior angle of pronotum, but not joining with inferior line; h) tibiae with apical ctenidium, only protibiae with 2–3 rows of subapical ctenidia; i) aedeagus subelliptical, or lenticular, with symmetrical and thin parameres (Fig. 9); j) female genital segment with large sternite, devoid of supplementary sclerites (Fig. 10H).

#### Key to species of the genus *Metolinus* Cameron from China (cf. Bordoni 2002).

**Table:** 

1	Elytra bicolorous, anterior 1/3 ochre, posterior 2/3 dark brown	*Metolinus shanicus* Bordoni
–	Elytra entirely dark brown, posterior margin sometimes narrowly yellowish	2
2	Dorsal integument with microsculpture of polygonal reticulum	3
–	Dorsal integument with distinct transverse microstriae	4
3	Lateral punctural row of pronotum with 3 punctures	*Metolinus hayashii* Bordoni
–	Lateral punctural row of pronotum with 6–8 punctures	*Metolinus xizangensis* sp. n.
4	Distance between punctures on dorsal surface of head ca. 5–6 puncture diameters	*Metolinus binarius* sp. n.
–	Distance between punctures on dorsal surface of head ca. 2–3 puncture diameters	5
5	Body rather small, shorter than 4.5 mm	*Metolinus planulatus* (Sharp, 1889)
–	Body medium sized, longer than 5 mm	6
6	Pronotum as long as head, abdominal terga III-VI entirely black	*Metolinus gardneri* (Cameron, 1945)
–	Pronotum longer than head, posterior margins of all abdominal terga broadly reddish	7
7	Sides of pronotum almost straight, slightly narrowed towards base	*Metolinus yunnanus* Bordoni, 2002
–	Sides of pronotum shallowly concave, at posterior angles about as wide as at anterior angles	*Metolinus emarginatus* sp. n.

### 1. Metolinus xizangensis

Zhou & Zhou sp. n.

urn:lsid:zoobank.org:act:7F979EE2-CBE3-4A82-874E-284C70120AB8

http://species-id.net/wiki/Metolinus_xizangensis

Fig. 10A–H Fig. 10-1A–E 

#### Type material.

Holotype: male, **CHINA: Xizang:** Cayu co.: Shangcayu (E 97.0994, N 28.7131), 1960 m, 21.VIII.2005, Wu Jie & Wang Xuejian collected (IZ-CAS); Paratypes: **CHINA: Xizang:** 6 males, 6 females, same data as holotype; 2 males, 1 female, same locality as holotype, 2000 m, 7.VIII.2005, Wu Jie collected (IZ-CAS).

#### Description.

*Measurement*. BL=7.9 mm, FL=3.8 mm, HL=1.2 mm, HW=0.93 mm, PL=1.3 mm, PW=0.90 mm, EL=1.3 mm, EW=1.1 mm.

Body nearly compressed and medium sized. Head, pronotum, mesoscutellum and elytra entirely dark brown. Abdomen dark brown, segment II and genital segments paler. Legs brown except femora obviously darker. Antennae entirely brown. Maxillary palpi and labial palpi light brown.

*Head*(Fig. 10-1A). Subrectangular (HL to HW ratio 1.3), tempora (behind eyes) slightly widened posteriorly, posterior angles rounded. Dorsal integument shiny, with extensive microsculpture composed of a mixture of transverse microstriae and polygonal reticulum, and with sparse, scattered with setiferous punctures of medium size, distance between punctures ca. 2 puncture diameters. With pair of frontal puncture on epistoma, 2 antennal punctures near antennal insertion, ocular puncture near medial margin of eye (ca. 3–4 puncture diameters to eye), temporal puncture at posterior 1/5 and occipital puncture at lateral 1/3; deflexed portion of tempora with same setiferous punctures and microstriae as on dorsal integument. Frontal furrows superficial and short, not longer than 1/2 of eye length. Ocular furrows of medium length, equal to eye length. Eye of medium size, nearly 1/3 of temple length (eye: temple = 0.23:0.68 mm), slightly protruding laterad. Epistoma protruding forwards, anterior margin subtruncated, dorsally flat and broad, as wide as 1/2 of eye length. Distance between antennal insertions ca. 0.32 mm, obviously wider than that from antenna to eye (ca. 0.26 mm). Ventral integument shiny, with polygonal reticulum, and with setiferous punctures as on dorsal integument, but obviously denser laterad. Mentum with a pair of setae inserted at anterior angle in addition to other irregularly scattered setae, submentum with 2 pairs of setae. Gular sutures fused at middle, and not separated at base of occiput. Gular plate devoid of punctures, with distinct transverse microstriae.

**Figure 10.**
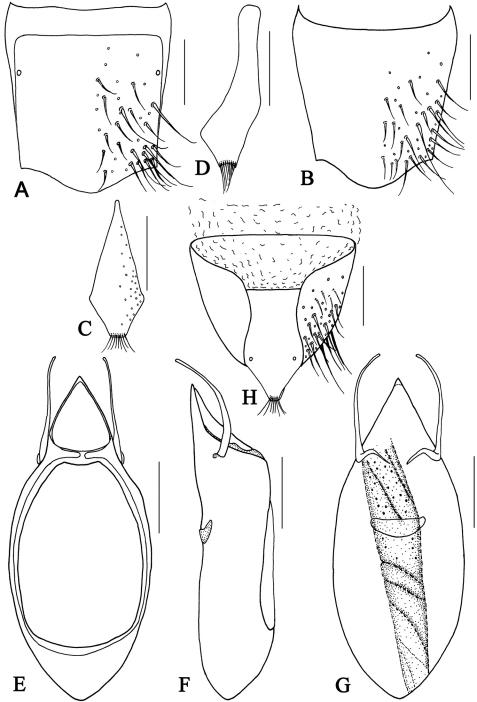
*Metolinus xizangensis* sp. n. **A** male tergite VIII **B** male sternite VIII **C** tergite of male genital segment **D** sternite of male genital segment **E** aedeagus, dorsal view **F** aedeagus, lateral view **G** aedeagus, ventral view **H** female genital segment, ventral view. Scale bars 0.15 mm.

**Figure 10-1.**
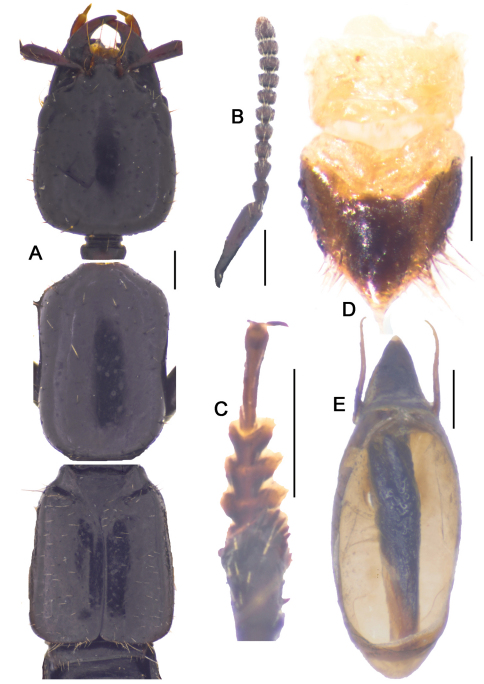
*Metolinus xizangensis* sp. n. **A** habitus of forebody **B** antennae **C** male protarsi; D. female genital segments, ventral view **E** aedeagus devoid of oval sclerite, ventral view. Scale bars 0.3 mm.

*Antennae* (Fig. 10-1B). Scape stout, thickened apically, longer than three subsequent antennomeres combined, ca. 0.45 mm; 2nd elongate, ca. 0.14 mm, distinctly longer than 3rd; 3rd globular, ca. 0.090 mm; 4th and 5th subequal, ca. 0.080 mm; last antennomere of medium length, ca. 0.15 mm, subequal to preceding 2 antennomeres combined.

*Mouthparts*. Labrum transverse and V-shaped bilobed, two lateral teeth subtruncated on anterior margin. Mandibles falciform, left one with two teeth on medial edge. Maxillary palpus elongate, with 3rd segment longest, last slender and aciculate. Labial palpus distinctly slender, with 2nd longest, last slender and aciculate.

*Neck*. Rather narrow (ca. 0.24 mm), approximately of 1/4 of head width.

*Pronotum*(Fig. 10-1A).Subrectangular, distinctly elongate (PL to PW ratio 1.4), of same length and width as head. Slightly widened anteriad, lateral margins concavely sinuate, anterior angles well defined, posterior angles broadly rounded. Integument shiny, extensively covered with oblique microstriae; with two rows of setiferous punctures on each side, admedian row consisting of 7–9 punctures and lateral row of 6–8 punctures obliquely arranged; hind angle puncture ca. 1–2 puncture diameters distant from lateral margin. Antesternal plate integrated and symmetrically shallowly concave medially; medial longitudinal suture missing, transverse suture at anterior 1/5 fine but visible. Prosternum with demarcated medial longitudinal carina on furcasternum, prosternal process (between anterior legs) triangularly projecting upwards. Mesoventrite extensively covered with transverse microstriae, medial longitudinal carina demarcated, process of mesoventrite triangularly protruding backwards. Metaventrite rather long, medial longitudinal keel sharp and obvious, without a fine furrow on posterior 1/3 of keel top; process of metaventrite subtruncated.

*Elytra*(Fig. 10-1A).Subrectangular, distinctly elongate (EL to EW ratio 1.2), of same length as pronotum, but obviously wider. Humeri well developed, lateral margins subparallel or slightly widened posteriorly, hind margin subtruncated. Integument shiny and flattened, without microsculpture, and with setiferous punctures arranged in several rows (more than 3 rows) on each elytron; deflexed portion of each elytron with 2–3 rows of sparse setiferous punctures.

*Legs*(Fig. 10-1C). First four segments of protarsi obviously dilated, heart shaped, bearing extremely dense clothing of white fine hairs ventrally, last tarsomere as long as III–IV combined. Last segment of both meso- and metatarsi longer than that of protarsi and about equal to length of II–IV combined. Tibiae with apical ctenidium, only protibia with 2–3 rows of subapical ctenidia.

*Abdomen*.Cylindrical, broadest at segment VI. Terga III–VII shiny, entire surface covered with distinct transverse microstriae, sparsely scattered with dot-like setiferous punctures, but denser laterobasally; each tergite with impunctate basal impression bearing more obvious transverse microstriae. All abdominal sterna shiny, with microstriae and setiferous punctures as those on terga.

*Male*.Tergite VIII entirely covered with setiferous punctures, except a narrow medial longitudinal impunctate band; posterior margins of tergite VIII and sternite VIII both arcuately protruding backwards (Fig. 10A, B). Tergite of genital segment symmetrical and small, with sharp base and subtruncated apex (Fig. 10C), *in situ* broadly exposed between pleurites. Pleurites of genital segment symmetrical, connected mediobasally. Sternite asymmetrical, with rounded base and more angular left side (Fig. 10D). Aedeagus pear-shaped and medium sized (Fig. 10-1E; Fig. 10E–G), basal bulbus ca. 1.50 mm long; median lobe triangular and long, ca. 1/3 of basal bulbus length. Parameres symmetrical and thin, ca. 1/3 of basal bulbus length. Internal sac with a cylindrical and spiral solid structure, of black color but base paler.

*Female*. Posterior margin of tergite VIII and sternite VIII broadly arcuate backwards. Genital segment medium sized (Fig. 10-1D; Fig. 10H), ca. 0.86 mm long. Sternite with subtruncated base. No additional sclerites attached on base of genital segment, except some membranous structures.

#### Distribution.

China (Xizang).

#### Etymology.

The specific epithet is the Chinese name (Pin-Yin) of the type locality.

#### Remarks.

This species could be distinguished from its congeners by microsculpture on head, the shape of the male genital segment (Fig. 10C, D) and the internal sac of the aedeagus (Fig. 10G).

### 2. Metolinus emarginatus

Zhou & Zhou sp. n.

urn:lsid:zoobank.org:act:3E4A7417-F19A-413C-A2E3-CFF0C59AC052

http://species-id.net/wiki/Metolinus_emarginatus

Fig. 11A–H Fig. 11-1A–E 

#### Type material.

Holotype: **CHINA:** **Sichuan:** male, Baoxing co. (E 102.8146, N 30.3681), Pujigou, 2450 m, 11.VIII.2003, Wu Jie collected (IZ-CAS); Paratypes: **CHINA:** **Sichuan:** 2 males, 4 females, same data as holotype.

#### Description.

*Measurement*.BL=5.8 mm, FL=2.9 mm, HL =0.84 mm, HW=0.78 mm, PL= 1.00 mm, PW=0.69 mm, EL=1.00 mm, EW=1.00 mm.

Body medium sized and nearly compressed. Body entirely dark brown, except each apical 1/3 of abdominal segment lighter. Legs dark brown, tarsi lighter. Antennae, maxillary palpi and labial palpi light brown.

*Head* (Fig. 11-1A). Subquadrate (HL to HW ratio 1.1), tempora (behind eyes) widened posteriorly, posterior angles rounded. Dorsal integument shiny, extensively covered with distinct transverse microstriae, and with sparse, scattered setiferous punctures of medium size, distance between punctures ca. 3 puncture diameters. With pair of frontal puncture on epistoma, 2 antennal punctures near antennal insertion, ocular puncture near medial margin of eye (ca. 3–4 puncture diameters from eye), temporal puncture at posterior 1/5 and occipital puncture at lateral 1/3; deflexed portion of tempora with same setiferous punctures and microstriae as on dorsal integument. Frontal furrows parallel and of medium length, longer than 1/2 of eye length. Ocular furrows deep and long, over eye length. Eye medium sized, nearly 1/3 of temple length (eye: temple =0.18:0.53 mm), slightly protruding laterad. Epistoma not protruding, anterior margin subtruncated, dorsally flat and broad, over 1/2 of eye length. Distance between antennal insertions ca. 0.23 mm, subequal to that from antenna to eyes (ca. 0.26mm). Ventral integument shiny, with same microstriae and setiferous punctures as on dorsal integument. Mentum with three pairs of setae inserted at each anterior angle in addition to other irregularly scattered setae, submentum with four pairs of setae. Gular sutures fused at middle, but separated at base of occiput. Gular plate devoid of punctures, but with distinct transverse microstriae.

**Figure 11.**
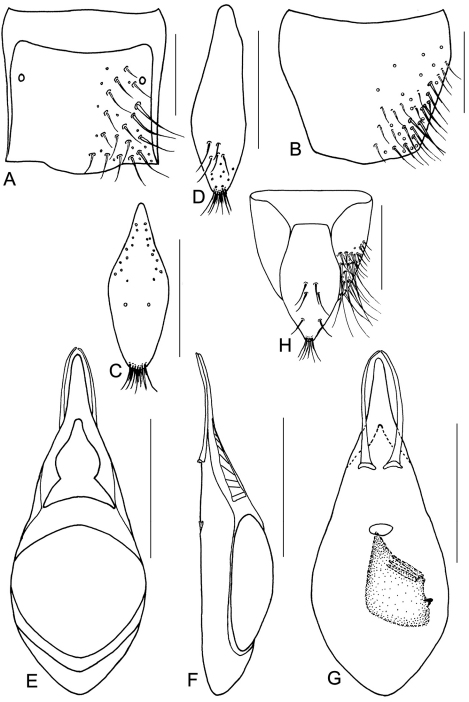
*Metolinus emarginatus* sp. n. **A** male tergite VIII **B** male sternite VIII **C** tergite of male genital segment **D** sternite of male genital segment **E** aedeagus, dorsal view **F** aedeagus, lateral view **G** aedeagus, ventral view **H** female genital segment, ventral view. Scale bars 0.15 mm.

**Figure 11-1.**
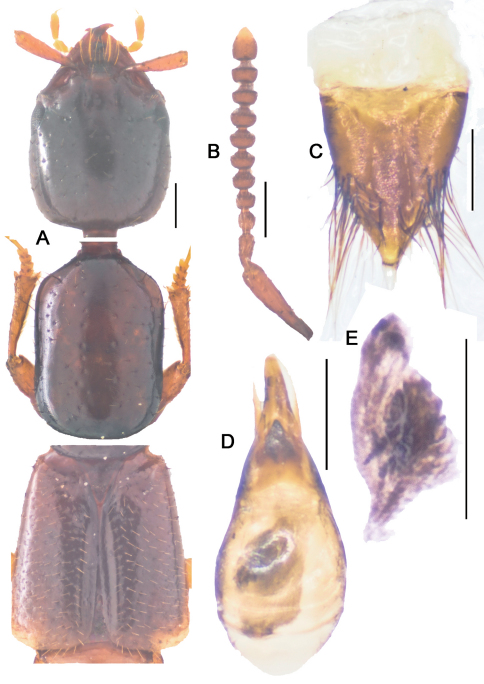
*Metolinus emarginatus* sp. n. **A** habitus of forebody **B** antennae **C** female genital segments, ventral view **D** aedeagus, dorsal view **E** inner sac. Scale bars 0.3 mm.

*Antennae* (Fig. 11-1B). Scape stout, thickened apically, longer than three subsequent antennomeres combined, ca. 0.45 mm; 2nd elongate, ca. 0.15 mm, distinctly longer than 3rd; 3rd globular, ca. 0.075 mm; 4th and 5th subequal, ca. 0.06 mm; last antennomere moderately long, ca. 0.15 mm, subequal to preceding 2 antennomeres combined.

*Mouthparts*. Labrum short and U-shaped bilobed, two subtruncated teeth on anterior margin. Mandibles falciform, left one with two teeth on medial edge. Maxillary palpus elongate, with 3rd segment longest, last slender and aciculate. Labial palpus distinctly slender, with 2nd longest, last slender and aciculate.

*Neck*. Rather narrow (ca. 0.24 mm), slightly wider than 1/4 of head width.

*Pronotum* (Fig. 11-1A).Subrectangular, distinctly elongate (PL to PW ratio 1.4), obviously longer than head, but of same width. Widened anteriad, lateral margins concavely sinuate; anterior angles well defined, posterior angles broadly rounded. Integument shiny, extensively with oblique microstriae; with two rows of setiferous punctures on each side, admedian row consisting of 7–9 punctures, lateral row of 6–8 punctures obliquely arranged; hind angle puncture ca. 1–2 puncture diameters distant from lateral margin. Antesternal plate integrated and not shallowly concave; medial longitudinal suture missing, transverse suture at anterior 1/5 fine but visible. Prosternum with demarcated medial longitudinal carina on posterior 1/5 of basisternum, anteriorly fused with prosternal process (between anterior legs). Mesoventrite extensively covered with transverse microstriae, medial longitudinal carina demarcated, process of mesoventrite arcuately protruding backwards. Metaventrite rather long, medial longitudinal keel sharp and obvious, with a fine furrow on posterior 1/2 of keel top; process of metaventrite subtruncated.

*Elytra* (Fig. 11-1A). Subquadrate, distinctly elongate (EL to EW ratio 1.0), of same length as pronotum, but distinctly wider. Humeri well developed, lateral margins widened posteriorly, hind margin convex. Integument shiny and flattened, without microsculpture, with setiferous punctures arranged in several rows (more than 3) on each side; deflexed portion of each elytron with 3–5 rows of punctures.

*Legs*.First four segments of protarsi obviously dilated, heart shaped, bearing extremely dense clothing of white fine hairs ventrally, last tarsomere as long as III–IV combined. Last segment of both meso- and metatarsi longer than that of protarsi and about equal to length of II–IV combined. Tibiae with apical ctenidium, only protibia with 2–3 rows of subapical ctenidia.

*Abdomen*.Cylindrical, broadest at segment VI. Terga III–VII shiny, entire surface covered with distinct transverse microstriae, with sparse, scattered, tiny setiferous punctures; each tergite with impunctate basal impression bearing more obvious transverse microstriae. All abdominal sterna shiny, with microstriae and setiferous punctures as those on terga.

*Male*.Tergite VIII entirely covered with setiferous punctures, except a narrow medial longitudinal impunctate band; posterior margin of tergite VIII (Fig. 11A) and sternite VIII (Fig. 11B) broadly arcuately protruding backwards,. Tergite of genital segment (Fig. 11C) symmetrical and medium sized, widest at midlength, with sharp base and rounded apex, in situ broadly exposed between pleurites. Pleurites of genital segment symmetrical, connected mediobasally. Sternite (Fig. 11D) asymmetrical, with rounded base and subtruncated right side. Aedeagus (Fig. 11-1DFig. 11E–G) pear-shaped and small sized, basal bulbus ca. 0.75 mm long; median lobe medium sized and sharply narrowed towards apex, ca. 1/3 of basal bulbus length. Parameres symmetrical and thin, ca. 1/3 of basal bulbus length. Internal sac (Fig. 11-1E; Fig. 11H) with brown and slightly sclerotized structure, composed of soft scales and 2–3 darker and transverse tubular-shaped structures medially.

*Female*. Posterior margin of tergite VIII and sternite VIII distinctly arcuate backwards. Genital segment (Fig. 11-1C)small, ca. 0.50 mm long. Sternite with subtruncated base. In addition, with some membranous structures attached to base of genital segment.

#### Distribution.

China (Sichuan).

#### Etymology.

The specific epithet is the Latin word *emarginatus* (emarginate) and refers to the shape of anterior margin of the epistoma.

#### Remarks.

Although the shape of the median lobe of the aedeagus seems similar to that of *Metolinus yunnanus* Bordoni, 2002 and *Metolinus loebli* Bordoni, 2002, the species may be distinguished by the longer parameres (Fig. 11E), different shape and composition of the internal sac (Fig. 11G), and by the tergite and sternite of the genital segment (Fig 11A, B).

### 3. Metolinus binarius

Zhou & Zhou sp. n.

urn:lsid:zoobank.org:act:EE6F836A-0CF1-48B6-B60E-C3A3DB7FEC94

http://species-id.net/wiki/Metolinus_binarius

Fig. 12A–H Fig. 12-1A–E 

#### Type material.

Holotype: **CHINA: Yunnan:** male, **Xishuangbanna Dai Autonomous Prefecture**, Menglun town (E 100.9876, N 22.1711), 860 m, 11.II.2004, Wu Jie collected (IZ-CAS); Paratypes: **CHINA: Yunnan: Xishuangbanna Dai Autonomous**, 5 males, 1 female, same data as holotype (IZ-CAS); 1ex., same data as holotype, except Wu Jie & Bai Dayuan collected (IZ-CAS); 2 males, 1 female, same locality as holotype, 560 m, 09.II.2004, Wu Jie collected (IZ-CAS); 1 male, 4 females, same locality as holotype, 550 m, 08.II.2004, Wu Jie & Bai Dayuan collected (IZ-CAS); 1ex., same locality as holotype, 730 m, 13.II.2004, Wu Jie collected (IZ-CAS); 1 female, same locality as holotype, 760 m, 10.II.2004, Wu Jie collected (IZ-CAS); Mengyang town: 1 female, Kungenaban 2nd village, 910 m, 08.X.2010, Zhou Yulingzi collected; 1 female, Mengla co., Nanman river, 857 m, 07.X.2010, Zhou Yulingzi collected (IZ-CAS).

#### Description.

*Measurement*. BL=5.0 mm, FL=2.3 mm, HL=0.62 mm, HW=0.53 mm, PL=0.75 mm, PW=0.51 mm, EL=0.83 mm, EW=0.72 mm.

Body medium sized and nearly compressed. Body entirely dark brown. Legs dark brown, tarsi lighter. Antennae, maxillary palpi and labial palpi light brown.

*Head* (Fig. 12-1A). Subrectangular (HL to HW ratio 1.2), tempora (behind eyes) obviously widened posteriorly, posterior angles rounded. Dorsal integument shiny, extensively covered with distinct transverse microstriae, and sparse, scattered setiferous punctures of medium size, distance between punctures ca. 5–6 puncture diameters. With pair of frontal puncture on epistoma, 2 antennal punctures near antennal insertion, ocular puncture near medial margin of eye (ca. 3–4 puncture diameters from eye), temporal puncture at posterior 1/5 and occipital puncture at lateral 1/3; deflexed portion of tempora with same setiferous punctures and microstriae as on dorsal integument. Frontal furrows superficial and short, slightly shorter than 1/2 of eye length. Ocular furrows of medium length, subequal to 1/2 of eye length. Eye of relatively large size, longer than 1/2 temporal length (eye: temple =0.18:0.29 mm), distinctly protruding laterad. Epistoma protruding forwards, anterior margin subtruncated, dorsally flat and broad, as wide as 1/2 of eye length. Distance between antennal insertions ca. 0.20 mm, obviously wider than distance from antenna to eyes (ca. 0.09 mm). Ventral integument shiny, with same microstriae and setiferous punctures as on dorsal integument. Mentum with a pair of setae inserted at each anterior angle in addition to other irregularly scattered setae, submentum with 2 pairs of setae. Gular sutures fused at middle, and not separated at base of occiput. Gular plate devoid of punctures, but with distinct transverse microstriae.

**Figure 12.**
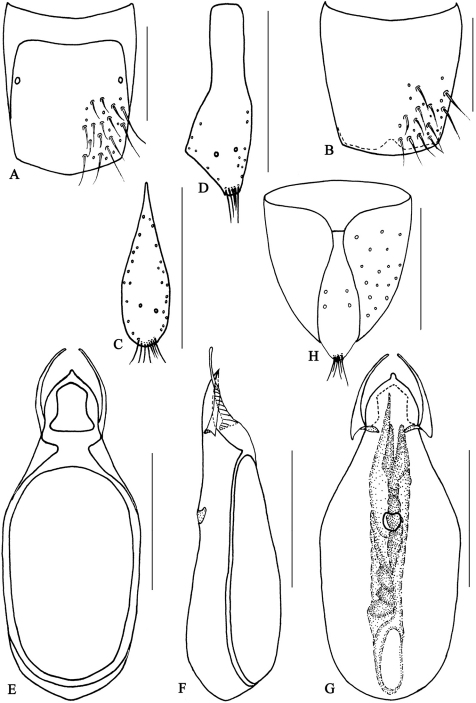
*Metolinus binarius* sp. n. **A** male tergite VIII **B** male sternite VIII **C** tergite of male genital segment **D** sternite of male genital segment **E** aedeagus, dorsal view **F** aedeagus, lateral view **G** aedeagus, ventral view **H** female genital segment, ventral view. Scale bars 0.15 mm.

**Figure 12-1.**
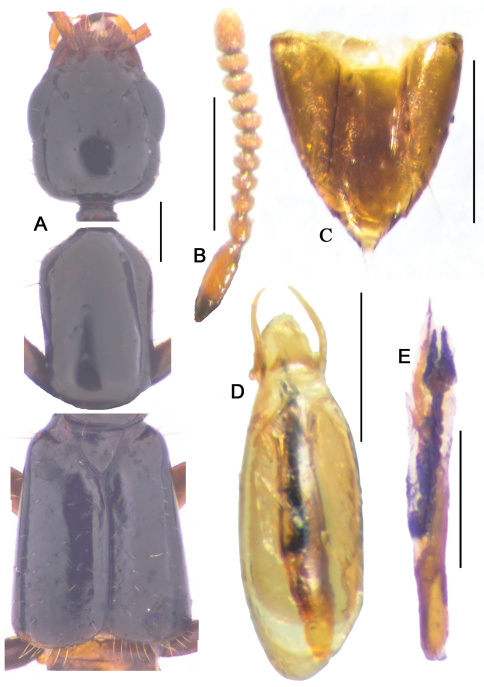
*Metolinus binarius*sp. n. **A** habitus of forebody **B** antennae **C** female genital segments, ventral view **D** aedeagus, dorsal view **E** inner sac. Scale bars 0.3 mm.

*Antennae*(Fig. 12-1B). Scape stout, thickened apically, longer than three subsequent antennomeres combined, ca. 0.23 mm; 2nd elongate, ca. 0.075 mm, distinctly longer than 3rd; 3rd globular, ca. 0.045 mm; 4th and 5th subequal, ca. 0.045 mm; last antennomere relatively long, ca. 0.11 mm, subequal to preceding 3 antennomeres combined.

*Mouthparts*. Labrum transverse and U-shaped bilobed, two lateral teeth subtruncated on anterior margin. Mandibles falciform, left medial edge bearing two teeth. Maxillary palpus elongate, with 3rd segment longest, last slender and aciculate. Labial palpus distinctly slender, with 2nd longest, last slender and aciculate.

*Neck*. Rather narrow (ca. 0.12 mm), approximately of 1/4 of head width.

*Pronotum* (Fig. 12-1A).Subrectangular, distinctly elongate (PL to PW ratio 1.5), obviously longer than head, but of same width. Slightly dilated anteriad, sides slightly concavely sinuate; anterior angles well defined, posterior angles broadly rounded. Integument shiny, extensively covered with oblique microstriae; with two rows of setiferous punctures on each side, admedian row consisting of 4–5 punctures, lateral row with 3–4 punctures obliquely arranged; hind angle puncture ca. 1–2 puncture diameters distant from lateral margin. Antesternal plate integrated, not concave; medial longitudinal and anterior transverse sutures both missing. Prosternum with demarcated medial longitudinal carina on furcasternum, prosternal process (between anterior legs) triangularly projecting upwards. Mesoventrite extensively covered with transverse microstriae, medial longitudinal carina missing, process of mesoventrite triangularly protruding backwards. Metaventrite rather long, medial longitudinal keel sharp and obvious, a fine furrow on posterior 1/3 of keel top; process of metaventrite subtruncated.

*Elytra*(Fig. 12-1A).Subrectangular, distinctly elongate (EL to EW ratio 1.2), obviously longer and wider than pronotum. Humeri well developed, lateral margins widened posteriorly, hind margin curved backwards. Integument shiny and flattened, without microsculpture. Each elytron with three rows of setiferous punctures, along suture, in middle and near lateral margin, with additional punctures irregularly scattered between them; deflexed portion of each elytron with 2–3 rows of sparse setiferous punctures.

*Legs*.First four segments of protarsi obviously dilated, heart shaped, bearing extremely dense clothing of white fine hairs ventrally, last tarsomere as long as III–IV combined. Last segment of both meso- and metatarsi longer than that of protarsi and about equal to length of II–IV combined. Tibiae with apical ctenidium, only protibia with 2–3 rows of subapical ctenidia.

*Abdomen*.Cylindrical, broadest at segment VI. Terga III–VII shiny, entire surface covered with distinct transverse microstriae, with sparse, scattered, tiny setiferous punctures, but denser laterobasally; each tergite with impunctate basal impression bearing more obvious transverse microstriae. All abdominal sterna shiny, with microstriae and setiferous punctures as those on terga.

*Male*. Tergite VIII entirely covered with setiferous punctures, except a narrow medial longitudinal impunctate band; posterior margin of tergite VIII and sternite VIII both broadly arcuate backwards (Fig. 12A, B). Tergite of genital segment (Fig. 12C) symmetrical and small, with sharp base and rounded apex, in situ broadly exposed between pleurites. Pleurites of genital segment symmetrical, connected mediobasally. Sternite (Fig. 12D) asymmetrical, with subtruncated base and more angular left side. Aedeagus (Fig. 12-1D; Fig. 12E–G) elliptical and small sized, basal bulbus ca. 0.80 mm long; median lobe distinctly shorter than 1/3 of basal bulbus length. Parameres symmetrical and thin, distinctly shorter than 1/3 of basal bulbus length. Internal sac (Fig. 12-1E; Fig. 12H) with well sclerotized structure, spines composed of larger tubular-shaped spines and dark brown complex spines, apical portion with a pair of spines and a lighter spine.

*Female*. Posterior margin of tergite VIII subtruncated, but sternite VIII broadly arcuate. Genital segment (Fig. 12-1C) small, ca. 0.45 mm long. Sternite with subtruncated base. Additional brown and transverse sclerite attached at base of genital segment.

#### Distribution.

China (Yunnan).

#### Etymology.

The specific epithet is derived from the Latin word *binarius* and refers to the pair of spines on the apical portion of the internal sac.

#### Remarks.

Although the large eyes and the number of punctures on the pronotum are similar to *Metolinus schulzvocki* Bordoni, 2003 and *Metolinus heuresilogus* Bordoni, 2002, it may be distinguished by the combined characters of the male genital segment (Fig. 12C, D) and the aedeagus (Fig. 12-1D; Fig. 12E–G).

### 4. Metolinus shanicus

Bordoni, 2002

http://species-id.net/wiki/Metolinus_shanicus

Fig. 13A–H Fig. 13-1A–E

Bordoni 2002: 375 (Type locality: Yunnan, Gaoligongshan Mts., 90 km W of Baoshan); Bordoni 2007: 71 (*Metolinus*; catalog)

#### Material examined.

2 males, 4 females, **CHINA: Yunnan:** Jingdong co.: Ailaoshan Field Station (E 98.2974, N 25.1119), 2486 m, 20.IX.2010, Zhou Yulingzi collected (IZ-CAS); 1 female, same locality as above, 21.IX.2010, Zhang Xi collected (IZ-CAS).

#### Description.

*Measurement*. BL=4.7 mm, FL=2.7 mm, HL= 0.74 mm, HW=0.66 mm, PL=0.87 mm, PW=0.60 mm, EL=0.99 mm, EW=0.78 mm.

Body small and nearly compressed. Head dark brown; pronotum, mesoscutellum, elytra and abdomen brown, except humeral portion (anterior 1/3 of elytra), posterior half of abdominal segment VII and entire VIII ochre. Legs entirely testaceous. Antennae, maxillary palpi and labial palpi castaneous.

*Head* (Fig. 13-1A). Subquadrate (HL to HW ratio 1.1), tempora (behind eyes) subparallel or slightly widened posteriorly, posterior angles rounded. Dorsal integument shiny, extensively covered with distinct transverse microstriae, and sparse, scattered setiferous punctures of medium size, distance between punctures ca. 4–5 puncture diameters. On each side symmetrically with frontal puncture on the epistoma, 2 antennal punctures near antennal insertion, ocular puncture near inner side of eye (ca. 3–4 puncture diameters from eye), temporal puncture at posterior 1/4 and occipital puncture at lateral 1/3; deflexed portion of tempora with same setiferous punctures and microstriae as on dorsal integument. Frontal furrows of medium length, ca. 2/3 of eye length, slightly curved and extending backward to same level of eye midlength. Ocular furrows of medium length, as long as eye length. Eye of medium size, nearly 1/3 of temple length (eye: temple =0.15:0.45 mm), and slightly protruding laterad. Epistoma protruding forwards, anterior margin subtruncated, dorsally flat and broad, as wide as 1/2 of eye length. Distance between antennal insertions ca. 0.23 mm, obviously wider than that from antenna to eyes (ca. 0.15 mm). Ventral integument shiny, with same microstriae and setiferous punctures as dorsal integument, except punctures deeper. Mentum with two pairs of setae inserted at anterior angles in addition to other irregularly scattered setae, submentum with 2 pairs of setae. Gular sutures fused at middle, not separated at base of occiput. Gular plate devoid of punctures, but with distinct transverse microstriae.

**Figure 13.**
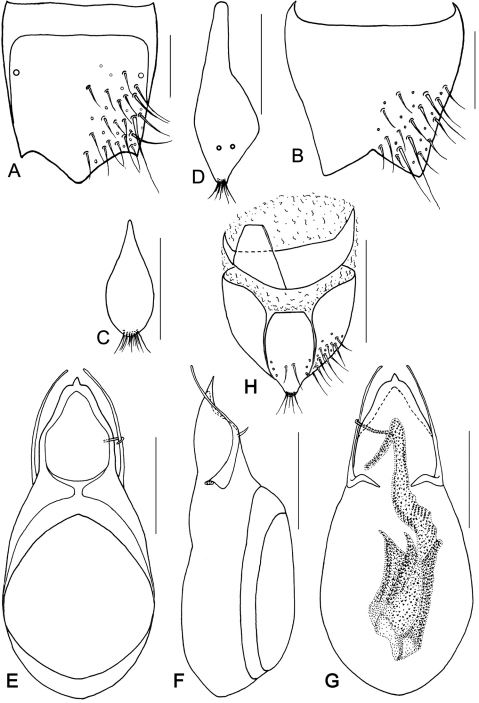
*Metolinus shanicus* Bordoni, 2002 **A** male tergite VIII **B** male sternite VIII **C** tergite of male genital segment **D** sternite of male genital segment **E** aedeagus, dorsal view **F** aedeagus, lateral view **G** aedeagus, ventral view **H** female genital segment, ventral view. Scale bars 0.15 mm.

**Figure 13-1.**
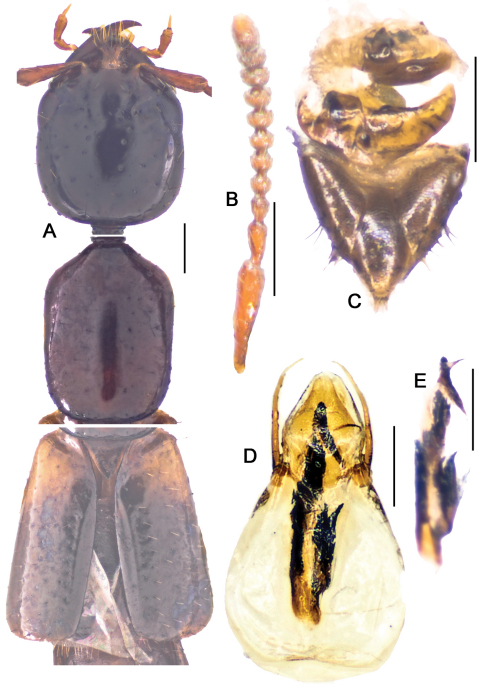
*Metolinus shanicus* Bordoni, 2002 **A** habitus of forebody **B** antennae **C** female genital segments, ventral view **D** aedeagus, dorsal view **E** inner sac. Scale bars 0.3 mm.

*Antennae* (Fig. 13-1B). Scape stout, thickened apically, longer than three subsequent antennomeres combined, ca. 0.33 mm; 2nd elongate, ca. 0.12 mm, distinctly longer than 3rd; 3rd globular, ca. 0.075 mm; 4th and 5th subequal, ca. 0.06 mm; last antennomere proportionately long, ca. 0.12 mm, subequal to preceding 3 antennomeres combined.

*Mouthparts*. Labrum transverse and V-shaped bilobed, with two subtruncated teeth on anterior margin. Mandibles falciform, left one with two teeth on medial edge. Maxillary palpus elongate, with 3rd segment longest, last slender and aciculate. Labial palpus distinctly slender, with 2nd longest, last slender and aciculate.

*Neck*. Rather narrow (ca. 0.17 mm), approximately of 1/4 of head width.

*Pronotum* (Fig. 13-1A).Subrectangular, distinctly elongate (PL to PW ratio 1.5), obviously longer than head but of same width as head. Slightly widened anteriad, lateral margins substraight; anterior angles well defined, posterior angles broadly rounded. Integument shiny, extensively covered with obliquely microstriae; with two rows of setiferous punctures on each side, admedian row consisting of 5–7 punctures, lateral row of 4–5 punctures obliquely arranged; hind angle puncture ca. 1–2 puncture diameters distant from lateral margin. Antesternal plate integrated and symmetrically shallowly concave medially; medial longitudinal suture missing, transverse suture at anterior 1/5 fine but observable. Prosternum with demarcated medial longitudinal carina on furcasternum, prosternal process (between anterior legs) triangularly projecting upwards. Mesoventrite extensively covered with transverse microstriae, medial longitudinal carina demarcated, process of mesoventrite triangularly protruding backwards. Metaventrite rather long, medial longitudinal keel sharp and obvious, without a fine furrow on keel top; process of metaventrite subtruncated.

*Elytra* (Fig. 13-1A).Subrectangular, distinctly elongate (EL to EW ratio 1.3), obviously longer and wider than pronotum. Humeri well developed, lateral margins subparallel, slightly widened posteriorly, hind margin subtruncated. Integument shiny and flattened, without microsculpture. Each elytron with three rows of setiferous punctures, along suture, in middle and near lateral margin, additional punctures scattered irregularly between them; deflexed portion of each elytron with 2–3 rows of sparse setiferous punctures.

*Legs*.First four segments of protarsi obviously dilated, heart shaped, bearing extremely dense clothing of white fine hairs ventrally, last tarsomere as long as III–IV combined. Last segment of meso- and metatarsi longer than that of protarsi and about equal to length of II–IV combined. Tibia with apical ctenidium, only protibia with 2–3 rows of subapical ctenidia.

*Abdomen*. Cylindrical, broadest at segment VI. Terga III–VII shiny, entire surface covered with distinct transverse microstriae, with sparse, scattered, round setiferous punctures, but denser laterobasally; each tergite with impunctate basal impression bearing more obvious transverse microstriae. All abdominal sterna shiny, with microstriae and setiferous punctures as those on terga.

*Male*. Tergite VIIIentirely covered with setiferous punctures, except a narrow medial longitudinal impunctate band; posterior margin of tergite VIII triangularly extended (Fig. 13A), that of sternite VIII triangularly emarginated (Fig. 13B). Tergite of genital segment (Fig. 13C) symmetrical, with sharp base and round apex, in situ broadly exposed between pleurites. Pleurites of genital segment symmetrical, connected mediobasally. Sternite (Fig. 13D) asymmetrical, with subtruncated base and more angular right side. Aedeagus (Fig. 13-1D; Fig. 13E–G) elliptical and medium sized, basal bulbus ca. 1.04 mm long; median lobe distinctly elongate, ca. 1/3 of basal bulbus length. Parameres symmetrical and of medium length, ca. 1/3 of basal bulbus length, thin and curved. Internal sac (Fig. 13-1E) with well sclerotized structure, some large spines on base, some small spines spirally arranged in middle, a long and thin flagellum extending out and intertwining right paramere in dorsal view.

*Female*. Posterior margin of tergite VIII and sternite VIII broadly arcuate backwards. Genital segment (Fig. 13-1C; Fig. 13H) small, ca. 0.40 mm long. Sternite with subtruncated base. Additional triangular-shaped sclerite attached to base of genital segment and folded by itself (Fig. 13-1C).

#### Distribution.

China (Yunnan).

#### Remarks.

This species may be distinguished from its congeners by the bicolorous elytra, the unique internal sac of aedeagus (Fig. 13G) and genital segment (Fig. 13C, D). However, the shape of the male tergite VIII of the specimens reported here slightly differs from the illustration given by Bordoni (2002: 374) which might reflect some degree of intraspecific variation.

### 5. Metolinus gardneri

(Cameron, 1945)

http://species-id.net/wiki/Metolinus_gardneri

Fig. 14A–H Fig. 14-1A–E

Cameron 1945: 68 (*Leptacinus*; Type locality: United Provinces: Dehra Dun, New Forest); Herman 2001: 3674 (*Leptacinus*; catalog); Bordoni 2002: 412 (*Metolinus*; characters; China, Thailand, Myanmar, Malaysia, Laos, Vietnam); Bordoni 2003a: 50 (*Metolinus*; Malaysia, W-Perak, 30 km SE Ipoh; Smetana 2004: 691 (*Metolinus*; catalog; Yunnan, India); Bordoni 2007: 71 (*Metolinus*; catalog).

#### Material examined.

**CHINA: Yunnan: Xishuangbanna Dai Autonomous Prefecture**, 1 female, 860 m, 11.II.2004, Wu Jie & Bai Dayuan collected (IZ-CAS); Yaoqu (E 101.6659, N 21.7007), 1 male, 1 female, 940 m, 19.II.2004, Wu Jie collected (IZ-CAS); Menglun town (E 100.9876, N 22.1711), 1ex., 620 m, 21.II.2004, Wu Jie & Zhang Jiaolin collected (IZ-CAS); Menglun town, 1 male, 730 m, 13.II.2004, Wu Jie collected (IZ-CAS); **Mengla co**., Longmen village, Xiaoniupeng (E 101.3252, N 21.3095), 1 female, 1035 m, 07.X.2010, Zhou Yulingzi collected (IZ-CAS); Mengyang town, 1 male, Kungenaban 2nd village (E 100.9876, N 22.1711), 910 m, 09.X.2010, collected by Lvliang (IZ-CAS).

#### Description.

*Measurement*. BL=4.9 mm, FL=2.7 mm, HL=0.80 mm, HW=0.63 mm, PL=0.90 mm, PW=0.63 mm, EL=0.89 mm, EW=0.69 mm.

Body small sized and nearly compressed. Head, pronotum, mesoscutellum and elytra entirely black. Abdomen dark brown, posterior margin of segment VII and VIII paler. Legs dark brown, tarsi lighter. Antennae brown except apical 1/2 of 11th segment yellowish. Maxillary palpi and labial palpi light brown.

*Head*(Fig. 14-1A). Subrectangular (HL to HW ratio 1.3), tempora slightly widened posteriorly, posterior angles rounded. Dorsal integument shiny, extensively covered with distinct transverse microstriae, and sparse, scattered setiferous punctures of medium size, distance between punctures ca. 3 puncture diameters. On each side symmetrically with frontal puncture on the epistoma, 2 antennal punctures near antennal insertion, ocular puncture near medial margin of eye (ca. 3–4 puncture diameters from eye), temporal puncture at posterior 1/5 and occipital puncture at lateral 1/3; deflexed portion of tempora with smaller setiferous punctures, and microstriae same as on dorsal integument. Frontal furrows superficial and short, shorter than 1/2 of eye length. Ocular furrows of medium length, subequal to eye length. Eye of medium size, slightly longer than 1/3 of temple length (eye: temple=0.18:0.42 mm), and slightly protruding laterad. Epistoma protruding forwards, anterior margin subtruncated, dorsally flat and broad, as wide as 1/2 of eye length. Distance between antennal insertions ca. 0.23 mm, obviously wider than distance from antenna to eyes (ca. 0.15 mm). Ventral integument shiny, with same microstriae and setiferous punctures as on dorsal integument. Mentum with two pairs of setae inserted at anterior angles in addition to other irregularly scattered setae, submentum with 3 pairs of setae. Gular sutures fused at middle, and separated at base of occiput. Gular plate devoid of punctures, but spread with distinct transverse microstriae.

**Figure 14.**
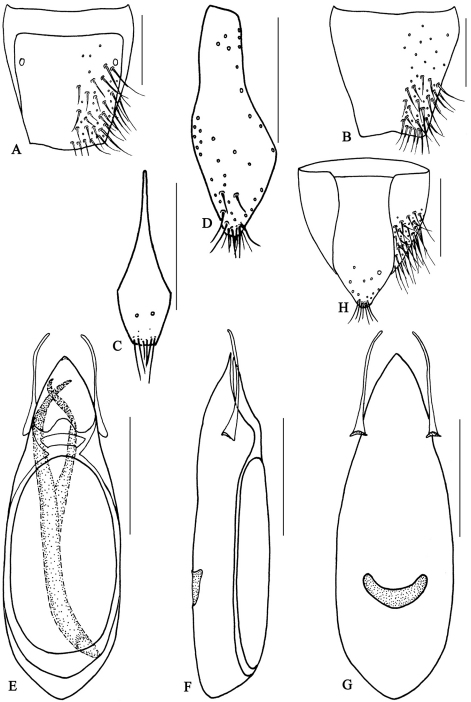
*Metolinus gardneri* (Cameron, 1945) **A** male tergite VIII **B** male sternite VIII **C** tergite of male genital segment **D** sternite of male genital segment **E** aedeagus, dorsal view **F** aedeagus, lateral view **G** aedeagus, ventral view **H** female genital segment, ventral view. Scale bars 0.15 mm.

**Figure 14-1.**
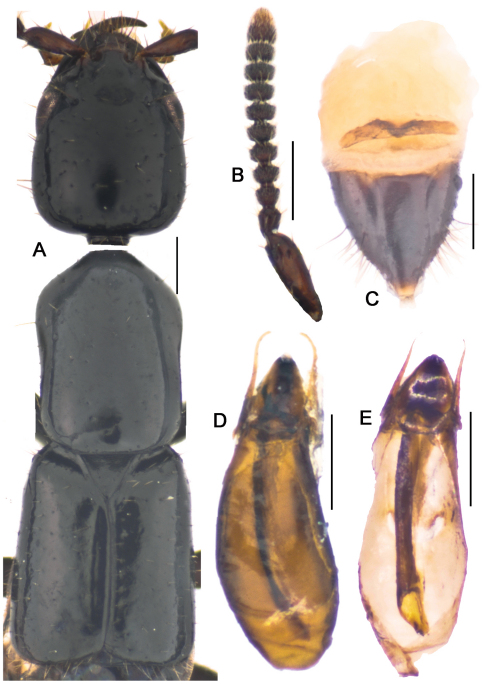
*Metolinus gardneri* (Cameron, 1945) **A** habitus of forebody **B** antennae **C** female genital segments, ventral view **D** aedeagus, dorsal view **E** inner sac. Scale bars 0.3 mm.

*Antennae*(Fig. 14-1B). Scape stout, thickened apically, longer than three subsequent antennomeres combined, ca. 0.30 mm; 2nd elongate, ca. 0.075 mm, distinctly longer than 3rd; 3rd globular, ca. 0.060 mm; 4th and 5th subequal, ca. 0.053 mm; last antennomere medium long, ca. 0.11 mm, as long as preceding 2 antennomeres combined.

*Mouthparts*. Labrum short and V-shaped bilobed, with two subtruncated teeth on anterior margin. Mandibles falciform, left with two teeth on medial edge. Maxillary palpus elongate, with 3rd segment longest, last slender and aciculate. Labial palpus distinctly slender, with 2nd longest, last slender and aciculate.

*Neck*. Rather narrow (ca. 0.17 mm), approximately of 1/4 of head width.

*Pronotum* (Fig. 14-1A). Subrectangular, distinctly elongate (PL to PW ratio 1.4), of same length and width as head. Slightly dilated anteriad, sides sinuate; anterior angles well defined, posterior angles broadly rounded. Integument shiny, extensively covered with oblique microstriae; with two rows of setiferous punctures on each side, admedian row consisting of 4–5 punctures, lateral row with 3–4 punctures straightly arranged; hind angle puncture ca. 1–2 puncture diameters distant from lateral margin. Antesternal plate integrated, not concave; medial longitudinal suture missing, transverse suture on anterior 1/5 fine but visible. Prosternum with demarcated medial longitudinal carina on furcasternum, prosternal process (between anterior legs) triangularly projecting upwards. Mesoventrite extensively covered with transverse microstriae, medial longitudinal carina missing, process of mesoventrite triangularly protruding backwards. Metaventrite rather long, medial longitudinal keel sharp and obvious, with a fine furrow on posterior 1/3 of keel top; process of metaventrite subtruncated.

*Elytra* (Fig. 14-1A). Subrectangular, distinctly elongate (EL to EW ratio 1.3), of same length and width as pronotum. Humeri well developed, lateral margins widened posteriorly, hind margin convex. Integument shiny and flattened, without microsculpture. Each elytron with three rows of setiferous punctures, along suture, in middle and near lateral margin, other punctures scattered irregularly between them; deflexed portion of each elytron with 2–3 rows of sparse setiferous punctures.

*Legs*.First four segments of protarsi obviously dilated, heart shaped, bearing extremely dense clothing of white fine hairs ventrally, last tarsomere as long as III–IV combined. Last segment of both meso- and metatarsi longer than that of protarsi and about equal to length of II–IV combined. Tibiae with apical ctenidium, only protibia with 2–3 of subapical ctenidia.

*Abdomen*.Cylindrical, broadest at segment VI. Terga III–VII shiny, entire surface covered with distinct transverse microstriae, with sparse, scattered, tiny setiferous punctures, but denser laterobasally; each tergite with impunctate basal impression bearing more obvious transverse microstriae. All abdominal sterna shiny, with microstriae and setiferous punctures as those on terga.

*Male*.Tergite VIII entirely covered with setiferous punctures, except for a narrow medial longitudinal impunctate band; tergite VIII with posterior margin broadly arcuate backwards (Fig. 14A), sternite VIII emarginated (Fig. 14B). Tergite of genital segment (Fig. 14C) symmetrical, with sharply pointed base and subtruncated apex, in situ broadly exposed between pleurites. Pleurites of genital segment symmetrical, connected mediobasally. Sternite (Fig. 14D) asymmetrical, with subtruncated base and more angular right side. Aedeagus (Fig. 14-1D; Fig. 14E–G) tubular-shaped and small sized, ca. 0.90 mm long; median lobe distinctly elongate, ca. 1/3 of basal bulbus length. Parameres symmetrical and thin, shorter than 1/3 of basal bulbus length. Internal sac (Fig. 14-1E) with well sclerotized structure, tubular-shaped, apical portion with two black and large spines.

*Female*. Posterior margin of tergite VIII subtruncated, that of sternite VIII subtruncated, and slightly transparent. Genital segment (Fig. 14-1C; Fig. 14H) small, ca. 0.58 mm long. Sternite with subtruncated base. Additional brown transverse sclerite attached to base of genital segment.

#### Distribution.

China (Yunnan); India, Thailand, Myanmar, Malaysia, Laos, Vietnam.

#### Remarks.

This species may be distinguished from its congeners by the few number of admedian punctural row of pronotum, the unique internal sac of the aedeagus (Fig. 14G) and genital segment (Fig. 14C, D).

### 6. Metolinus hayashii

Bordoni, 2002

http://species-id.net/wiki/Metolinus_hayashii

Bordoni 2002: 359 (Type locality: S-China, Jizushan Mts, 40 km N of Binchuan; 25 51N, 100 34E, 2600 m; 19–21.VI.1995).

#### Material examined.

None.

#### Distribution.

China (Yunnan).

### 7. Metolinus planulatus

(Sharp, 1889)

http://species-id.net/wiki/Metolinus_planulatus

Sharp 1889: 252 (*Leptacinus*; Type locality: Hitoyoshi; Kuma Kuni); Bernhauer and Schubert 1914: 294 (*Leptacinus*; catalog); Shibata 1983: 73 (*Leptacinus*; check list of the family Staphylinidae of Japan. III); Herman 2001: 3681 (*Leptacinus*; Japan); Bordoni 2002: 426 (*Metolinus*; characters; China, Fujian, Shaowu, Tachuland; 24.IV.1942); Smetana 2004: 691 (catalog; Fujian, Sichuan, Japan); Bordoni 2007: 71 (*Metolinus*; catalog).

#### Material examined.

None.

#### Distribution.

China (Fujian); Japan.

### 8. Metolinus yunnanus

Bordoni, 2002

http://species-id.net/wiki/Metolinus_yunnanus

Bordoni 2002: 405 (Type locality: S-China, Yunnan, Heishui, 35 km N Lijiang; 27 13N, 100 19E; 1–19.VII.1992); Bordoni 2007: 71 (*Metolinus*; catalog)

#### Material examined.

None.

#### Distribution.

China (Yunnan).

**Figure 15.**
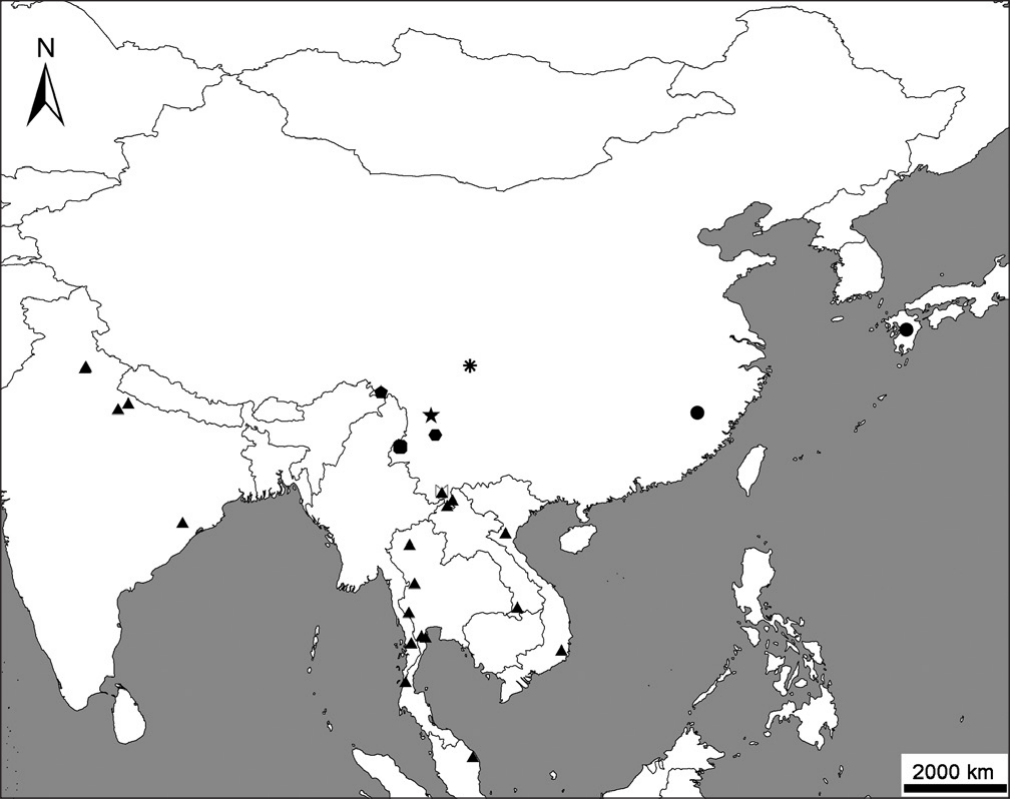
Geographical distribution of species of the genus *Metolinus* Cameron in China. XXX M. gardneri (Cameron, 1945); XXX M. planulatus (Sharp, 1889); XXX M. binarus sp. n.; XXX M. emarginatus sp. n.; XXX M. hayashii Bordoni, 2002; XXX M. shanicus Bordoni, 2002; XXX M. xizangensis sp. n.; XXX M. yunnanus Bordoni, 2002.

## Supplementary Material

XML Treatment for Metolinus

XML Treatment for Metolinus xizangensis

XML Treatment for Metolinus emarginatus

XML Treatment for Metolinus binarius

XML Treatment for Metolinus shanicus

XML Treatment for Metolinus gardneri

XML Treatment for Metolinus hayashii

XML Treatment for Metolinus planulatus

XML Treatment for Metolinus yunnanus
